# A New Dinoflagellate Genome Illuminates a Conserved Gene Cluster Involved in Sunscreen Biosynthesis

**DOI:** 10.1093/gbe/evaa235

**Published:** 2020-11-04

**Authors:** Eiichi Shoguchi, Girish Beedessee, Kanako Hisata, Ipputa Tada, Haruhi Narisoko, Noriyuki Satoh, Masanobu Kawachi, Chuya Shinzato

**Affiliations:** 1 Marine Genomics Unit, Okinawa Institute of Science and Technology Graduate University, Onna, Okinawa, Japan; 2 Department of Genetics, The Graduate University for Advanced Studies, SOKENDAI, Mishima, Shizuoka, Japan; 3 Center for Environmental Biology and Ecosystem Studies, National Institute for Environmental Studies, Tsukuba, Ibaraki, Japan; 4 Atmosphere and Ocean Research Institute, The University of Tokyo, Kashiwa, Chiba, Japan

**Keywords:** Symbiodiniaceae, *Durusdinium trenchii*, WGS, MAAs, GMC oxidoreductase

## Abstract

Photosynthetic dinoflagellates of the Family Symbiodiniaceae live symbiotically with many organisms that inhabit coral reefs and are currently classified into fifteen groups, including seven genera. Draft genomes from four genera, *Symbiodinium*, *Breviolum*, *Fugacium*, and *Cladocopium*, which have been isolated from corals, have been reported. However, no genome is available from the genus *Durusdinium*, which occupies an intermediate phylogenetic position in the Family Symbiodiniaceae and is well known for thermal tolerance (resistance to bleaching). We sequenced, assembled, and annotated the genome of *Durusdinium trenchii*, isolated from the coral, *Favia speciosa*, in Okinawa, Japan. Assembled short reads amounted to 670 Mb with ∼47% GC content. This GC content was intermediate among taxa belonging to the Symbiodiniaceae. Approximately 30,000 protein-coding genes were predicted in the *D. trenchii* genome, fewer than in other genomes from the Symbiodiniaceae. However, annotations revealed that the *D. trenchii* genome encodes a cluster of genes for synthesis of mycosporine-like amino acids, which absorb UV radiation. Interestingly, a neighboring gene in the cluster encodes a glucose–methanol–choline oxidoreductase with a flavin adenine dinucleotide domain that is also found in *Symbiodinium tridacnidorum*. This conservation seems to partially clarify an ancestral genomic structure in the Symbiodiniaceae and its loss in late-branching lineages, including *Breviolum* and *Cladocopium*, after splitting from the *Durusdinium* lineage. Our analysis suggests that approximately half of the taxa in the Symbiodiniaceae may maintain the ability to synthesize mycosporine-like amino acids. Thus, this work provides a significant genomic resource for understanding the genomic diversity of Symbiodiniaceae in corals.

**Significance**

Dinoflagellates of the family Symbiodiniaceae include coral symbionts and have been well studied. Analyses from whole-genome sequencing of several genera have been reported, but no genome is available from the genus *Durusdinium*. Here, we report the draft genome of *Durusdinium trenchii* from the coral, *Favia speciosa*. The genomic analysis of this thermotolerant species shows that a cluster of genes for biosynthesis of mycosporine-like amino acids (MAAs), which absorb UV radiation, is conserved between *Symbiodinium*, an early-diverging lineage, and *Durusdinium*, which occupies an intermediate phylogenetic position in the Family Symbiodiniaceae. Both genera reportedly enhance thermal tolerance of corals. If coral bleaching is triggered by high solar radiation, a dinoflagellate capacity for MAA biosynthesis may contribute to bleaching resistance.

## Introduction

Symbiotic dinoflagellates of the Family Symbiodiniaceae, are keystone photosynthetic organisms in coral reef ecosystems (LaJeunesse et al. 2018). The diversity of symbiotic dinoflagellate populations and their relationships with hosts have been analyzed and discussed (Baker 2003; Coffroth and Santos 2005; Coffroth et al. 2006; Pochon and Gates 2010; Green et al. 2014; Pochon et al. 2014). Dinoflagellate populations in stony corals, which form modern reefs, have attracted particular attention (Abrego et al. 2009; Thornhill et al. 2014; Shoguchi et al. 2020), because breakdown of coral-dinoflagellate symbiosis causes coral bleaching, decimating coral reef communities (coral holobionts) (Weis et al. 2008; Stat and Gates 2011).

Although many reasons have been discussed for collapse of this symbiotic relationship (Nakamura and van Woesik 2001; Iguchi et al. 2012), the main trigger is likely rising surface seawater temperatures (SSTs) caused by climate change (Weis et al. 2008). Possible bleaching has been also reported in other hosts, such as giant clams (Mies 2019). Recently, discussions of coral bleaching due to increasing SSTs have focused on heat-tolerant species of the genus *Durusdinium* (Symbiodiniaceae) (a member of previous clade D), because genetic variability of dinoflagellates in corals is thought to be a major factor in the bleaching phenomenon (Berkelmans and Van Oppen 2006; Stat and Gates 2011; Lesser 2019). Horizontal transmission types of symbiosis may be more adaptive than vertical transmission types (Weis et al. 2001; Yamashita et al. 2013; Hidaka 2016; Yuyama et al. 2018). Coral holobionts resulting from coral-*Durusdinium* symbiosis may be better adapted to rising SSTs than other types of coral holobionts.

*Durusdinium* includes heat-tolerant strains (Rowan 2004; Stat and Gates 2011). A metabolic analysis of cultured Symbiodiniaceae showed that *D. trenchii* has a low level of the sterol metabolite, C_29_ Stanol 2, suggesting metabolic differences among members of the family Symbiodiniaceae (*Symbiodinium microadriaticum*, *Symbiodinium psygmophilum*, and *B. minutum*) (Klueter et al. 2015). A recent report on effects of light and thermal stress indicates that the pan-tropical species, *D. trenchii*, is more thermotolerant than others so far examined (*S. microadriaticum*, *B. minutum*, and *Cladocopium goreaui*) (Lesser 2019). In addition, the heat-stress response of *D. trenchii* was compared between free-living and symbiotic cells and transcriptional activity in symbiotic dinoflagellates was drastically altered by thermal stress (Bellantuono et al. 2019).

Genomes of several taxa within the Symbiodiniaceae have been deciphered (Shoguchi et al. 2013, 2018; Aranda et al. 2016; Liu et al. 2018; Li et al. 2020) and genome evolution of this family has been discussed (González-Pech et al. 2019). However, no *Durusdinium* genome is available and the genetic basis for thermal tolerance remains unknown (Baker 2003; Weber and Medina 2012; Hidaka 2016). To provide a genomic resource for *Durusdinium*, we isolated *D. trenchii* from the coral, *F. speciosa*, in Okinawa, which will be useful for analyzing *Durusdinium* in coral holobionts.

One of the early-diverging lineages of the family Symbiodiniaceae is the genus *Symbiodinium* (LaJeunesse et al. 2018), which includes species having the ability to synthesize MAAs (Banaszak et al. 2000). Both *Symbiodinium* and *Durusdinium* have been known to enhance thermal tolerance of holobionts (Reynolds et al. 2008; Kemp et al. 2014; Aihara et al. 2016). The genome of *Symbiodinium tridacnidorum* has a cluster of genes for enzymes involved in MAA biosynthesis (Shoguchi et al. 2018). On the other hand, species in later-diverging groups (*Breviolum* and *Cladocopium*) appear not to have this metabolic pathway, as no MAA gene cluster has been found in their genomes. Therefore, a draft genome of *Durusdinium*, one of an intermediate group of seven genera in the family Symbiodiniaceae, may help to clarify when the ability to synthesize MAAs was lost during diversification of the Symbiodiniaceae. To explore the genetic background of the coral symbiont, *Durusdinium*, here we examined the genome and associated transcriptomes to determine whether *Durusdinium* also has this gene cluster.

## Materials and Methods

### Biological Materials

The culturable dinoflagellate, *D. trenchii*, is harbored by the coral, *F. speciosa*, in Okinawa, Japan. A single cell of *D. trenchii* was isolated using a glass-micropipette in May 2012. The Nagoya Protocol was not applicable to the dinoflagellate. The established culture strain is available as NIES-2907 in the Microbial Culture Collection at the National Institute for Environmental Studies (NIES) in Tsukuba (https://mcc.nies.go.jp). Cloned *Durusdinium* cells for nucleotide sequencing were basically maintained as previously described (Shoguchi et al. 2018). The culture medium included artificial seawater containing 1× Guillard’s (F/2) marine-water enrichment solution (Sigma–Aldrich) and soil extract (Provasoli et al. 1957). A 25 °C incubator for culturing was maintained on a 12 h-light/12 h-dark regime at an illumination of ∼20 μmol m^−2^ s^−1^ (Beedessee et al. 2015).

### Nucleotide Sequencing and Assembly

Genomic DNA from clonal cultures was extracted using phenol-chloroform and cetyltrimethylammonium bromide (Shoguchi et al. 2013) and was used for Illumina library construction (supplementary table S1, Supplementary Material online). Libraries were sequenced using a HiSeq 2500 (Illumina) and paired-end reads were assembled de novo with Platanus (Kajitani et al. 2014) and Newbler. Assembled data were combined (Nishitsuji et al. 2020). Scaffolding with mate-pair information was carried out using SSPACE (ver. 3.0) (Boetzer et al. 2011). With Gapcloser, gaps inside scaffolds were closed with paired-end data. Finally, data were polished using Pilon (ver. 1.22) (Walker et al. 2014). The completeness of the assembled genome was evaluated by the recovery of 458 CEGMA and 303 BUSCO genes from the genome of *D. trenchii* (Parra et al. 2007; Simão et al. 2015; Beedessee et al. 2020). Total RNA for transcriptome sequencing was isolated from cultured cells at 25 °C, as described previously (Shoguchi et al. 2013). Two libraries (the difference of culture time at 0 and 3 days) were constructed following the manufacturer’s protocol and were sequenced using a HiSeq 2500. De novo assembly was performed using Trinity (Grabherr et al. 2011).

### Gene Prediction and Annotation

RNA-seq reads were mapped to a soft-masked genome using STAR (Dobin et al. 2013) for passage to the BRAKER2 pipeline (Hoff et al. 2016). UTR and gene model prediction were performed with Augustus (v3.2.3) (Stanke et al. 2008). Intron and exon hints were generated with STAR (Dobin et al. 2013) and BLAT (Kent 2002), respectively, and were used to make final gene predictions using a modified version of Augustus (v3.2.3) (Stanke et al. 2008; Shoguchi et al. 2013). The final set of predicted proteins was annotated against UniProt (Magrane and UniProt Consortium 2011) and PFAM (Punta et al. 2012) where hits larger than 1e^−5^ were discarded. Putative contaminant sequences were identified basically following Chen et al. (2020). First, short scaffolds (<1 kb) were removed (Shoguchi et al. 2018). To find contaminant sequences, 45 scaffolds (>10 kb) with high GC content (>55%) were manually checked using a genome browser (Koyanagi et al. 2013). Twelve scaffolds predicted genes with introns that were supported by transcriptomes or had similarities to *S. microadriaticum* proteins (BlastX, E-value <10^−20^) in the NCBI database (https://pubmed.ncbi.nlm.nih.gov). About 33 scaffolds were removed as putative contaminant sequences.

### Molecular Phylogenetic Tree and Protein Structure Predictions

Molecular phylogenetic analysis of the demethyl-4-deoxygadusol (DDG) synthase family was performed as described in our previous study (Shoguchi et al. 2018). Protein sequences of glucose–methanol–choline (GMC) oxidoreductases in the molecular phylogenetic analysis of Sorigué et al. (2017) and some proteins with GMC domains were collected from the NCBI database (https://pubmed.ncbi.nlm.nih.gov; last accessed July 21, 2020). Those and Symbiodiniaceae proteins with GMC domains were aligned with MAFFT (Katoh and Standley 2013). Molecular phylogenetic analysis was carried out using Bayesian inference with MrBayes v.3.2 (Ronquist et al. 2012), as previously described (Beedessee et al. 2019). Trees were visualized using Figtree (http://tree.bio.ed.ac.uk/software/figtree/). I-TASSER was used for 3 D prediction (Zhang 2008).

## Results and Discussion

### Draft Genome of *Durusdinium*

Three genomic libraries with insert sizes ranging from 500 bp to 19 kb were constructed from the cloned *Durusdinium* (supplementary table S1, Supplementary Material online). Short read sequencing (2 × 101 bp) produced ∼76 Gb of total sequencing data, which were assembled into a total length of 695 Mb. Thirty-three scaffolds, which were likely to be contaminant sequences, were removed from the initial assembly (supplementary table S2, Supplementary Material online). The final draft genome of *D. trenchii* (version 1.0) had a total length of 670.4 Mb with a scaffold N50 of 97.5 kb (table 1). Completeness of the *D. trenchii* genome was checked using CEGMA (Parra et al. 2007) and BUSCO (Simão et al. 2015). The 48% (145/303 BUSCO genes) hits on the *D. trenchii* proteins was comparable to other reported dinoflagellate genomes (44–71%) (supplementary fig. S1, Supplementary Material online). The GC content of the draft genome was 47.4%, comparable to GC contents of *Symbiodinium* (∼50%) and *Cladocopium* (∼44%) (table 1).

**Table 1:** Genomic Compositions of Seven Genomes of the Family Symbiodiniaceae

	*Durusdinium trenchii*	*Symbiodinium microadriaticum* ^a^	*Symbiodinium tridacnidorum* ^b^	*Breviolum minutum* ^c^	*Fugacium kawagutii v3* ^d^	*Cladocopium goreaui* ^e^	*Cladocopium* sp. (C92)^b^
A total assembled length of assembly (Mb)	670.43	808.24	766.65	615.52	936.98	1,027.79	704.77
G + C content (%)	47.4	50.5	49.9	43.6	45.5	44.8	43.0
No. of genes	30,054	49,109	69,018	41,925	45,192	35,913	65,832
Average length of genes (bp)	15,030	12,898	8,834	11,959	7,242	6,967	8,192
No. of exons per gene	19.6	21.8	13.4	19.6	12.6	10.0	11.3
Average length (bp) of exons	90	110	105	100	126	176	130
Average length (bp) of introns	704	505	561	499	479	575	622

a Aranda et al. (2016).

b Shoguchi et al. (2018).

c Shoguchi et al. (2013).

d Li et al. (2020).

e Liu et al. (2018).

### Genome Annotations

Two RNA-seq libraries were constructed and sequenced. Reads of 2 × 134 bp produced ∼11 Gb of total sequencing data (supplementary table S1, Supplementary Material online). The de novo assembly produced 64,183 contigs with a GC content of ∼55%, similar to that of clade D from reported transcriptomes (González-Pech et al. 2017). Using transcriptome data as hints, 30,054 protein-coding genes were predicted (table 1), a number comparable to that of *C. goreaui* (Liu et al. 2018), but less than some other dinoflagellate genomes. A recent report indicated that a consistent gene-prediction approach is crucial for comparative genomic analysis, suggesting the difficulties of computational gene prediction for dinoflagellate genomes (Chen et al. 2020). Therefore, long-read transcriptomic data from various conditions are likely to be needed in future comparative genomic studies. Assembled genomic and transcriptomic data and annotation information are accessible from the following genome browser: https://marinegenomics.oist.jp/gallery (Koyanagi et al. 2013). The 28S rDNAs and ITS2 sequences (TRINITY_DN41397_c4_g2_i5) from the assembled sequences corresponded to the nuclear ribosomal ITS1/5.8S/ITS2 (KJ019889) in *D. trenchii* LaJeunesse sp. nov. (LaJeunesse et al. 2014, 2018), confirming that the clone is *D. trenchii*.

### Gene Cluster for Sunscreen Biosynthesis

**Fig. 1:**
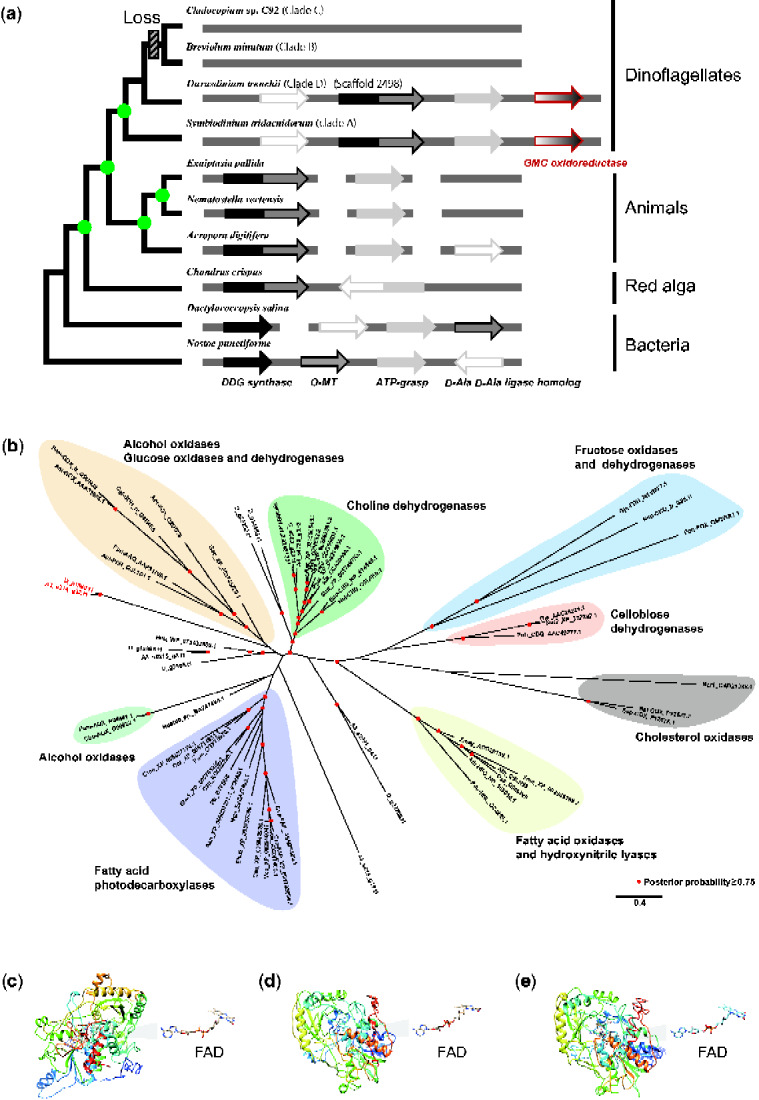
The dinoflagellates, *Symbiodinium tridacnidorum* and *Durusdinium trenchii*, both possess a probable gene cluster for MAA biosynthesis. (*a*) A gene cluster in the *D. trenchii* genome and a potential evolutionary scenario for MAA biosynthesis in the family Symbiodiniaceae. Topology of the tree is based on the phylogenetic tree of the DDG synthase family with bootstrap support >90%, as shown in green circle. The detail is shown in supplementary figure S2, Supplementary Material online. The positions of clades B and C with no MAA biosynthetic gene cluster are assumed based upon previous 28S rDNA phylogenies (Shoguchi et al. 2018). (*b*) A molecular phylogeny of GMC family enzymes showing evolutionary relationships of the proteins. Proteins from the neighboring *GMC*  *oxidoreductase* in (*a*) the MAA biosynthetic gene cluster are shown in red. Genes for choline dehydrogenase are encoded in dinoflagellate genomes and others in the Symbiodiniaceae are unclassified enzymes in the GMC family. (*c–e*) 3D structures of the enzymes and their use of flavin adenine dinucleotide (FAD) as a cofactor were predicted using I-TASSER (Zhang 2008). (*c*) Fatty acid photodecarboxylase (FAP), a light-activated enzyme from *Chlorella variabilis*. (*d*) g1386 of *D. trenchii*. (*e*) s314_g32.t1 of *S. tridacnidorum*.

Analysis of the *S. tridacnidorum* (previously *Symbiodinium* sp. clade A3) genome identified a gene cluster for enzymes involved in MAA biosynthesis in Shoguchi et al. (2018). In addition, comparative analysis suggested that orthologs of these genes have been lost in the common ancestor of *Breviolum* and *Cladocopium*. To determine whether such losses occurred in the *Durusdinium* lineage, we performed BLAST and Pfam domain searches. Scaffold 2498 of the draft assembly contained a gene cluster for MAA biosynthesis, expression of which was supported by transcriptomic data (fig. 1*a*). Moreover, gene order was conserved between *S. tridacnidorum* and *D. trenchii*. The orthologous relationship between *S. tridacnidorum* and *D. trenchii* was confirmed in molecular phylogenetic analysis of the DDG synthase family (supplementary fig. S2, Supplementary Material online). In addition, we found that the neighboring gene to *D-Ala D-Ala ligase homolog* on the 3′ side of the cluster encoded an enzyme resembling the GMC oxidoreductase family. The homolog was also found in the genome of *S. tridacnidorum* and is located adjacent to the MAA gene cluster (fig. 1*a*), suggesting syntenic conservation of metabolic genes among members of the Symbiodiniaceae (Liu et al. 2018).

The predicted ligase had domains for the GMC oxidoreductase family (GMC_oxred_N of PF00732 and GMC_oxred_C of PF05199), which includes proteins having diverse catalytic activities. A molecular phylogeny of GMC oxidoreductases indicated that this one does not belong to a subfamily with known functions and that it may constitute a sister group of alcohol oxidases and glucose oxidases and dehydrogenases (fig. 1*b*). Recently, it has been shown that GMC oxidoreductases in algae include photoenzymes (Sorigué et al. 2017; Björn 2018), which have the light-capturing flavin adenine dinucleotide (FAD) as a cofactor (fig. 1*c*). Using I-TASSER software, prediction of the 3D structure of Symbiodiniaceae GMC oxidoreductases showed that they likely also carry FAD (fig. 1*d* and *e*), suggesting the possibility of a photoenzyme (Sorigué et al. 2017; Björn 2018). Future studies may clarify the relationship between light intensity and MAA biosynthesis. Coral genomes also have genes for MAAs (Shinzato et al. 2011, 2014), but they do not seem to have GMC oxidoreductase. If coral bleaching is triggered by high SSTs and insolation (Lesser 2019), the Symbiodiniaceae capacity for MAA biosynthesis may contribute to bleaching resistance.

## Supplementary Material

Supplementary data are available at *Genome Biology and Evolution* online.

## Supplementary Material

evaa235_Supplementary_Data
