# Supplementary material for: A New Dinoflagellate Genome Illuminates a Conserved Gene Cluster Involved in Sunscreen Biosynthesis
Source: Genome Biol Evol. 2020 Nov 4;13(2):evaa235. doi: 10.1093/gbe/evaa235 (PMC7875005; doi:10.1093/gbe/evaa235)
Supplement: evaa235_Supplementary_Data [file evaa235_supplementary_data.pdf]

Supplementary Table S1

Summary of Illumina data used for assembling the genome and transcriptomes of *Durusdinium trenchii*

Nucleic acid types	Methods, library*	Total sequences (Gb)	Number of reads (million)	Read length (bp)
DNA	Illumina Hiseq, 500-600 bp PE	74.88	777.21	2 x 101
DNA	Illumina Hiseq, 1.5-2.0 kbp MP	0.92	10.82	2 x 101
DNA	Illumina Hiseq, 12.0-19.0 kbp MP	0.12	1.47	2 x 101
RNA	Illumina Hiseq, ~200 bp PE	11.20	85.04	2 x 134

*PE: Paired End; MP: Mate Pair

Supplementary Table S2

Putative bacterial sequences in an initial assembly of the *Durusdinium trenchii* genome

Scaffold	Length	GC%
sc0000001	3818788	55.4467
sc0000003	3374619	68.7074
sc0000005	2905589	58.1473
sc0000006	1941893	58.9728
sc0000007	1845437	59.2217
sc0000008	1650576	59.1353
sc0000010	1160861	61.6839
sc0000011	1072958	59.8257
sc0000012	994447	62.0804
sc0000014	829373	62.1216
sc0000015	780951	59.2596
sc0000016	701201	58.6964
sc0000018	635352	59.8572
sc0000034	446913	61.623

sc0000036	442620	58.428
sc0000077	341569	60.8129
sc0000219	256152	61.0548
sc0000408	207333	60.8107
sc0000623	180561	60.4769
sc0000774	162685	58.8038
sc0000789	162869	59.1904
sc0000905	153482	59.4388
sc0001436	119183	57.8482
sc0001971	97912	58.8812
sc0002081	95206	61.7364
sc0004185	51425	59.1347
sc0006929	23952	61.7443
sc0007229	21979	56.3267
sc0007438	20711	60.2916
sc0009319	11254	63.2664
sc0009346	11246	55.182
sc0009517	10603	60.4357
sc0009620	10291	55.1335

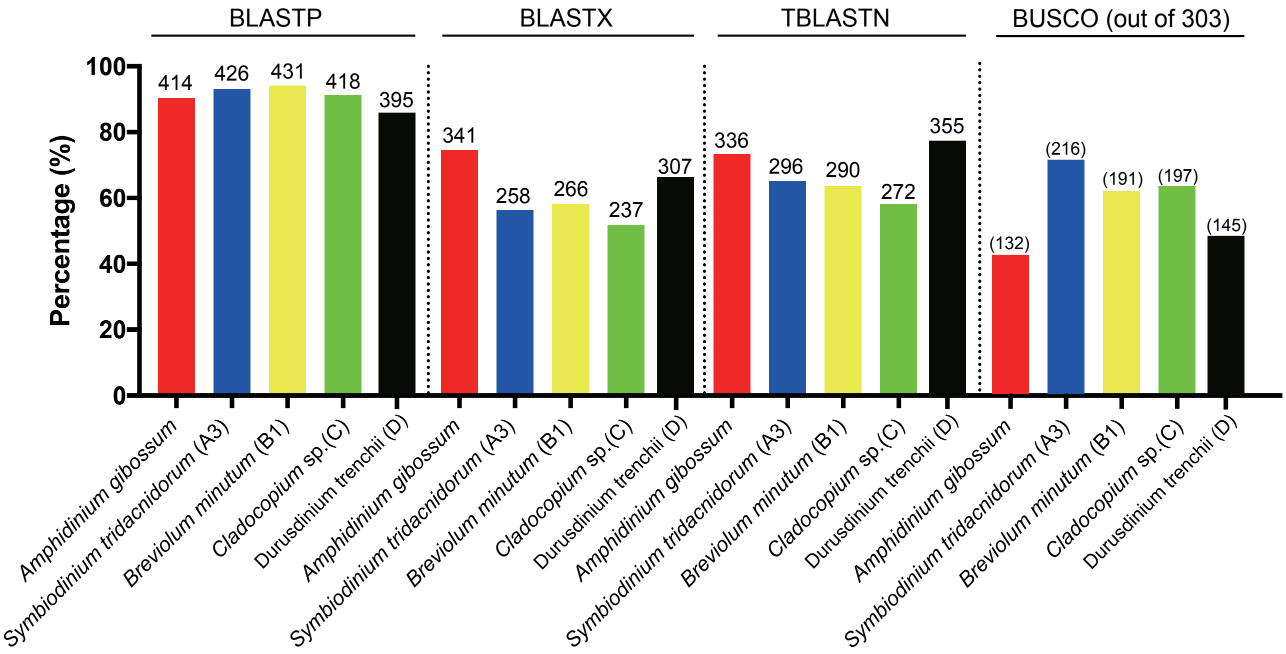

Supplementary Fig. S1.

CEGMA and BUSCO scores for the *Durusdinium trenchii* (clade D) genome and other available genomes of dinoflagellates. Recoveries of 458 CEGMA genes, based on several BLAST analyses using BLASTP (for predicted proteins) and on BLASTX and TBLASTN (for genome scaffolds), are shown (Beedessee et al. 2020). Recoveries of 303 BUSCO genes using protein mode and the Eukaryota_odb9 dataset are compared. Numbers in brackets represent recovered BUSCOs gene numbers.

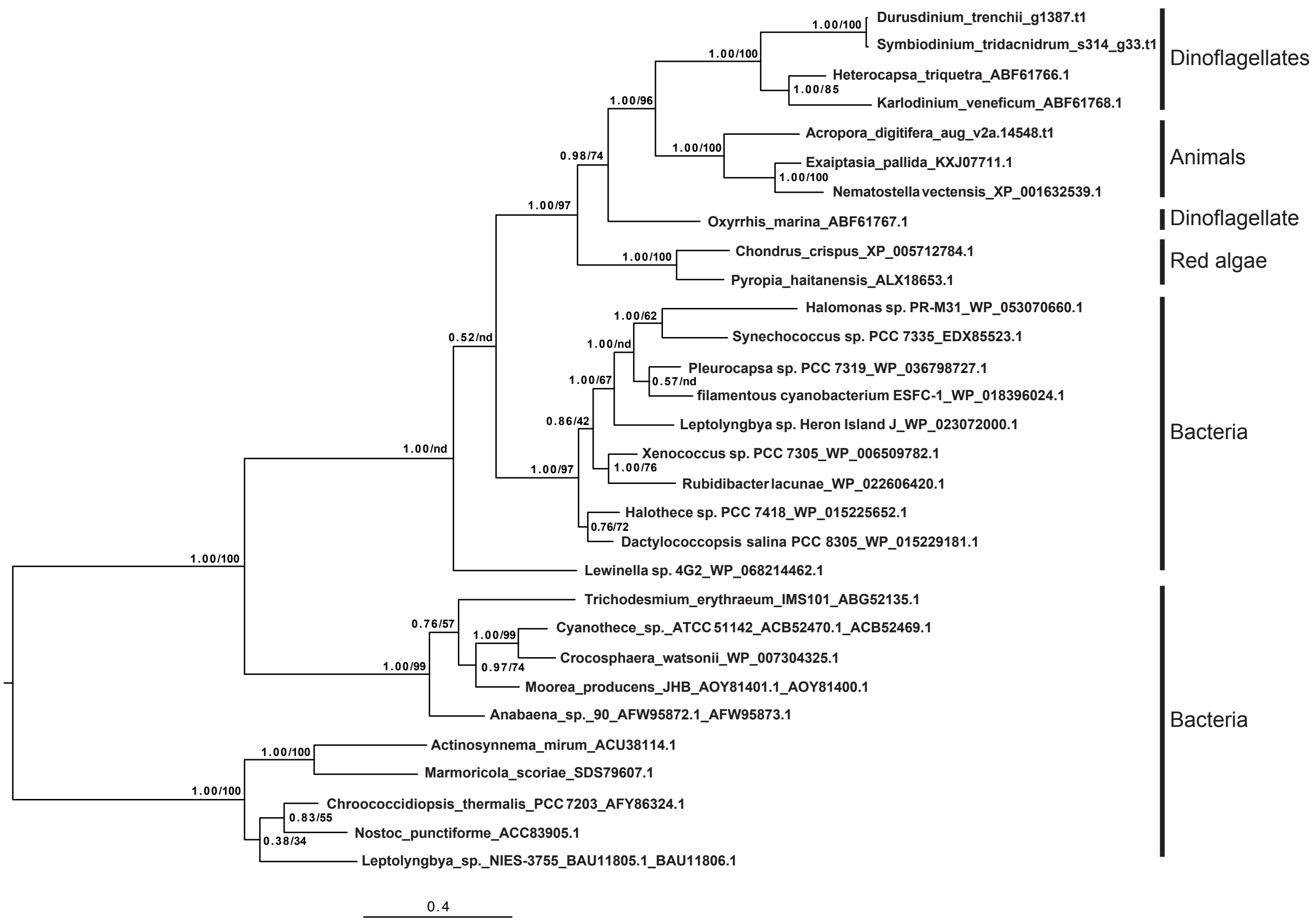

Supplementary Fig. S2.

A molecular phylogenetic tree of the DDG (dimethyl 4-deoxygadusol synthase) family by Bayesian inference and maximum likelihood. Posterior probabilities (MrBayes) and bootstrap values (LG+G model and RAxML) are shown at nodes. Some values were not determined by different topologies (nd).
